# Low-Stress Mechanical Property Study of Various Functional Fabrics for Tactile Property Evaluation

**DOI:** 10.3390/ma11122466

**Published:** 2018-12-05

**Authors:** Melkie Getnet Tadesse, Ladislav Nagy, Vincent Nierstrasz, Carmen Loghin, Yan Chen, Lichuan Wang

**Affiliations:** 1 College of Textile and Clothing Engineering, Soochow University, 1 Shi Zi Street, Suzhou 215006, China; melkie.tadesse@hb.se (M.G.T.); yanchen@suda.edu.cn (Y.C.); 2 Department of Textile Materials Technology, The Swedish School of Textiles, University of Boras, 501 90 Boras, Sweden; vincent.nierstrasz@hb.se; 3 Faculty of Textiles, Leather & Industrial Management, Gheorghe Asachi Technical University of Iasi, 53, D. Mangeron Blv., 700050 Iasi, Romania; cloghin@tex.tuiasi.ro; 4 Department of Clothing, Technical University of Liberec, CZ 46117 Liberec, Czech Republic; ladislav.nagy@tul.cz

**Keywords:** functional fabrics, low-stress mechanical properties, tactile property, KES

## Abstract

Functional finishing brings an alteration on the mechanical and surface properties of textile materials and henceforth influences the tactile properties. In this work, Kawabata evaluation systems (KES) for fabrics were utilized to notice the changes in the tactile properties of fabrics resulting from different finishing types such as inkjet printing, screen printing, and coating. The effects of functional finishing on the fabric’s tactile property were inconsistent with reference to the course of decrease or increase being dependent on the types of finishes. The findings showed that KES can be employed as a promising tool to sort out the suitable functional finishing types in terms of tactile properties. Amongst the implemented finishing types, inkjet printing offered superior tactile properties with respect to tensile energy (softness), shear rigidity, compressional softness, bending stiffness (drapability), and surface properties. The KES results confirmed that low-stress mechanical properties are strongly associated with the tactile property and might assist as a quality profile data source for guaranteeing the production and development of a virtuous quality product. The result encourages further utilization of the KES for functional fabric tactile property evaluation.

## 1. Introduction

Recent research are witnessed that functional textiles are on a big demand on the domains of e-textiles progress. Functional fabrics are those fabrics entirely designed for explicit uses such as conducting electricity, providing information which is significant to the user, creating a signal, getting rid of moisture, repelling water, and provide insulating purposes. They are the most integral parts of e-textiles. Moreover, these functional textiles might interrupt the comfort of the wearer if their process of manufacturing is not restricted and managed concisely. Therefore, the effect of different functional finishing shall be investigated with regards to the tactile properties. As functionality can be introduced either mechanically (such as weaving) or chemically (such as printing), the effects on the comfort of the fabric during wearing could not be neglected whether the smart fabric touches the entire or some parts of the body. The fact is that the inclusion materials or processes can influence the wellbeing of the user.

The tactile property has a strong connection to the low-stress mechanical properties of textile-based goods intended for wearing [1]; the authors described that the peak density and the weave type can influence the thermal comfort of the textile-based fabrics. Behera (2007) [2] identified linen fabrics from its blends by studying the low-stress mechanical properties in terms of tactile properties and handle. A previous study [3] measured the tactile properties of cotton fabrics using KES and found a strong relationship amongst the fabric structural parameters and the tactile properties of the product which sequentially affect the comfort of the final product.

The tactile properties of clothing materials comprise the typical features of clothing quality as asserted in a past paper [4]. Clothing quality is indebted to switch the decision-making of consumers during purchasing of textile goods as revealed previously [5]. The tactile properties of the textile materials can be explained by measuring the mechanical properties objectively and quantitatively. The objective measurement of textile fabrics was traced back to 1930 [6], where the author described the fabric handle from the perspective of the stiffness, smoothness, and softness using ordinary objective measurement tools. Softness, which is the most criterion of the fabric’s handle, has been determined by measuring the mechanical properties using KES [7]. Smoothness, the other imperative factor used for the determination of the fabric tactile property, has been described with respect to surface contours using stereo vision techniques objectively [8]. Past research [9] identified the changes in the handle of the fabrics perceived due to fire retardant finishes using a simple extraction method.

After the birth of the objective evaluation of the handle of textile products, other methods such as KES [10], fabric assurance by simple testing (FAST) [11], and fabric touch tester (FTT) [12] have been realized in-depth as the effective methods for measuring the handle of the textile-based product by measuring the mechanical properties of the fabrics. It is true that there are some limitations to the aforementioned instruments. In the 1980s, Kawabata and his coworkers [13] developed Kawabata’s evaluation system (KES), a sophisticated equipment to measure the low-stress mechanical properties of various kinds of polymeric fabrics such as tensile, shearing, bending, compression, thickness, weight, surface, and frictional properties of the textile-based materials to determine the handle of the product.

Fabric objective evaluation has been used for the determination of the tactile property of the ordinary textile fabrics. A comparative study of silk, cotton, and polyester fabrics has been introduced previously [14], where the effect of finishing on the mechanical properties has been identified using KES. The effects of finishing have been explored using objective measurement [15]. They tested the mechanical properties of the fabrics which are related to fabric make-up and worsted finishing by means of FAST and found that the low-stress mechanical properties are critical for tailoring and can contribute to understand the stability of worsted finishing.

There are a few alternative methods to measure the mechanical properties of the fabrics objectively. A previous work [16] has employed an Instron testing instrument and they managed to measure nine mechanical properties effectively, seven parameters less than that of KES where 17 parameters can be explored. The concentrated loading method has been put in place to study the low-stress mechanical properties of woven fabrics [17]. Almadar found that measuring the tensile properties can be validated using such methods while the shearing properties can only be estimated. All the lengthy procedures and the cost of the instruments, KES systems are the best alternative to measure the low-stress mechanical properties effectively as mentioned previously [13]. This is because KES not only predicts human perception but also understands the perception of primary hands.

Nevertheless, there has been lots of work performed on the on determination of ordinary textiles objective handle pursued by special instruments, no suitable instruments or standard techniques have been established for functional fabrics. Since functional fabrics are relatively new products when compared to ordinary summer and winter suits, hand evaluation techniques for the functional fabrics does not yet exist, although the tactile properties of the functional fabric are as crucial as that of the ordinary fabrics. However, objective evaluation for technical textiles has been performed in a similar way to that of the ordinary textiles as mentioned previously [18]; they identified the effect of plasma and fire retardant finishing on the surface and stiffness properties of the treated fabric using KES. This is the turning point to use the objective measurement for functional fabrics. Furthermore, the ordinary and functional fabrics vary only on the finishing techniques applied to the functional fabric; therefore, they share similar perception when touched. Finishing techniques can influence the handle of the textile-based material as described previously [19]. Mohar and Postle easily identified the effect of fabric finishing, dry-cleaning, and steam pressing by measuring the low-stress mechanical properties of the fabrics. Therefore evaluation of the effects of various functional finishing techniques applied to textile products is required. Based on this assumption, objective measurements were performed on the functional textile fabrics with the same principle with that of the ordinary fabrics to observe the effect of the functional finishing on tactile properties.

In this paper, we extend the application of objective hand evaluation of the ordinary textile fabrics to functional fabrics using KES instruments finished with various types of treatments including coating, inkjet printing, and screen printing. We investigated the utilization of KES measurement principles in quality control and inspection in the production and development of functional textile fabrics. As per the author’s knowledge, this kind of attempt has been rarely or not yet performed for the quality control of the functional fabrics.

## 2. Materials and Methods

### 2.1. Material Preparation

Five functional textile fabrics were carefully prepared for this study. Sample details are shown in Table 1. Polyester fabric, a substrate used to produce the functional fabrics, with a fabric weight of 158 g/m^2^, 30 ends cm^−1^, and 22 picks cm^−1^ was scoured and heat-set by the supplier (Almedahl-Kinna AB, Kinna, Sweden) and used as the control sample (sample 7), see the details in a past work [20].

### 2.2. Methods

Mechanical properties under low-load regions were measured and examined using KES FB-Auto (Kato Tech Co., Ltd., Kyoto, Japan) with their details listed in Table 2. Fifteen low-stress mechanical properties were measured in both the warp and the weft ways excluding the compressional property; the average was reported.

**Figure 1:**
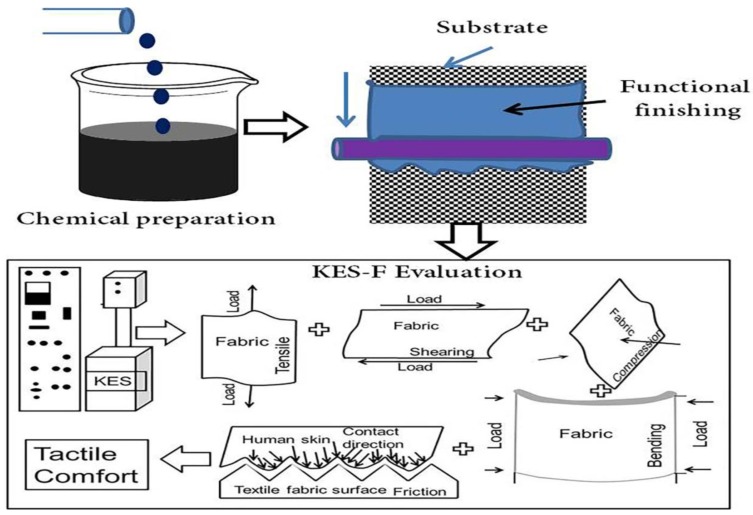
Chart which shows the low-stress mechanical properties measurement principle and process flow in relation to the tactile properties of the functional fabrics.

Figure 1 shows the overall mechanical properties that could be measured under low-load regions. Each sample fabric was prepared by following specific standards according to the previously published resources and presented and further used for Kawabata evaluation. The size of each sample was according to the Kawabata evaluation standard.

## 3. Results and Discussion

The most critical step in developing smart or functional fabrics is having a desirable quality value with respect to human satisfaction which relies upon recognizing the need for an immense definition and investigation of tactile properties. An integrated and all-inclusive approach to evaluate the functional fabrics quality is unquestionably very paramount. Among these approaches, measurement and interpretations of low-stress mechanical properties of functional fabrics are the main detrimental steps ahead. Here, this methodology is presented to interpret the tactile properties of the functional fabrics. The mechanical properties such as tensile, shearing, bending, compression, and surface friction under low-load conditions were measured and analyzed by utilizing Kawabata’s evaluation systems for fabrics (KES) under the test conditions mentioned previously [13].

### 3.1. Correlations between Mechanical Properties

In order to observe the strengths of the relationships between the mechanical properties of the functional fabrics, correlation coefficients were computed and analyzed. The results of the computation are shown in Table 3.

As shown in the table, mechanical properties are highly correlated to each other and most are significant at the 99% confidence level (*p* < 0.01). The mean deviation of surface roughness SMD gets highly significant correlation value (*p* < 0.001) to tensile properties. This means surface physical roughness is the uppermost significant parameter in the evaluation of functional fabric’s hand with respect to the tensile properties. The conflicting idea to this closure is the fact that we realized that the frictional coefficient MIU had few correlations to the mechanical properties. Functional finishing may affect the two surface properties in a different way such that finishing techniques brought a change in the surface roughness and hence the surface roughness of the samples increased. Therefore, the slipperiness property (MIU) may be concealed by the surface roughness. The negative and positive sign in the correlation coefficients are the signs of the direction of correlations between mechanical parameters. For instance, the correlation between linearity LT and tensile energy WT is −0.87 while between LT and tensile resilience RT is 0.88. Therefore, taking this result into account, we can claim that as the tensile linearity of the curve in the tensile test increases, the amount of tensile energy wasted reduces. On the other hand, as the linearity of the curve increases, the tensile resilience of the product increases consequently. The coefficient of correlation elucidated that satisfying associations were observed between the mechanical properties.

### 3.2. Effect of Functional Finishing on Low-Stress Mechanical Properties

There are abundant situations requiring great effort in acquiring an agreeable fabric handle requirements of functional fabrics treated with different finishing chemicals and practices. Tactile property tests using KES measurement instruments might determine the functional fabric handle, which is a critical quality index for users by the minute making the purchasing decisions. The KES system includes four distinct tools for testing tensile, shearing, bending, compression, surface friction, and constructional (thickness and weight) properties which are defined by a total of 17 parameters. The test results of these parameters are presented below.

#### 3.2.1. Tensile Properties

**Figure 2:**
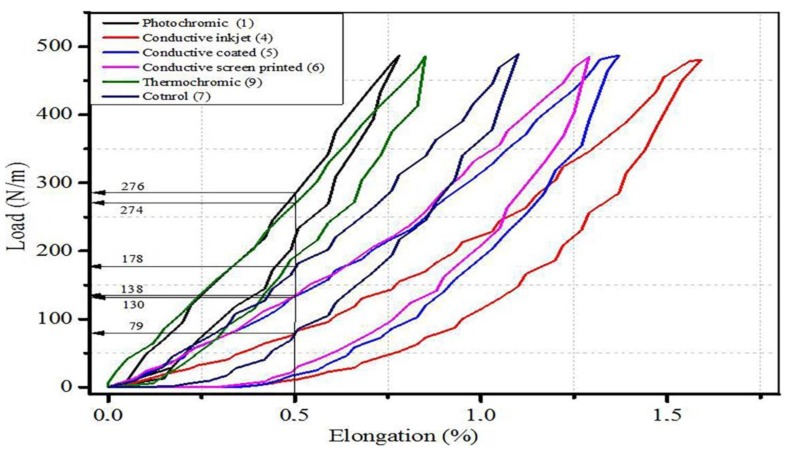
A load–elongation curve of functional fabrics (warp way). These measurements were taken to compare the KES results of different functional fabrics treated with different application methods. They have the same substrate material; polyester fabric with 158 gsm. The load–elongation curve clearly indicates the effects of functional finishing on extensibility.

Figure 2 shows a typical load–elongation curve of samples during loading–unloading conditions where the tensile behaviors of the samples are thoroughly dependent on the finishing treatments. Thus, the tensile performance associated to the tactile property of the fabric can be elucidated via these aspects. For instance, to extend sample 4 required a total load of ~79 N/m, while a force more than 3.5 times than sample 4 (~276 N/m) was required to extend a photochromic sample at 0.5% elongation. This could be attributed to the utilizations of different chemical reagents, application methods, and drying curing process for each sample. The conductive inkjet solution (Sample 4) constitutes PEDOT-PSS, glycerol, water, and surfactant. On the other hand, the photochromic paste (Sample 1) constitutes photochromic dye and varnish. This indicates that KES measurement helps to detect the differences in tensile behaviors of samples while applying different finishing parameters of each sample. The inkjet-printed sample is found to be more extensible than the control fabric. This may be due to wet effect due to accompaniments of water and glycerol on the functional fabric which makes the sample more extensible. Fabric extensibility in the initial loading range was extreme; this serves as a piece of information that the fabrics are more comfortable during wearing. The higher the extensibility is, the better the quality of the textile product in terms of tactile properties. Therefore, the load–elongation curves of the functional fabric might provide evidence regarding the tactile property of the product.

Therefore, based on the load–elongation cure outputs, inkjet-printing (conductive), coating, screen printing, and inkjet printing (thermochromic) produces the best to the least extensibility property, respectively. However, it is very challenging to conclude the final tactile property of the functional fabrics using only the load–elongation curve as the sensory property is a very complex phenomenon and the load–elongation curve is drawn in the warp direction only.

**Figure 3:**
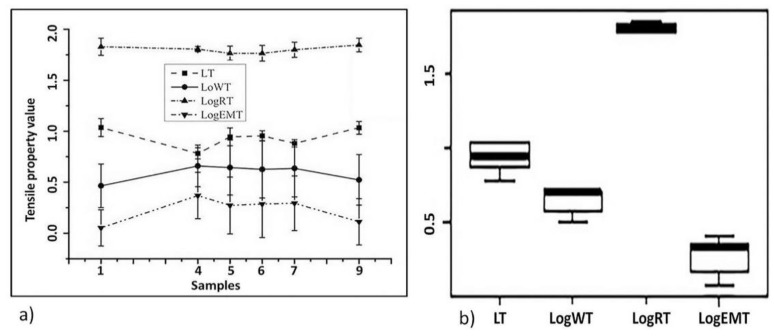
(**a**) The tensile properties measurement result and (**b**) the variability of the tensile measurements within the samples using box plot. The logarithmic values of some of the mechanical properties are used to draw the figure for scale purposes only. The polyester fabric treated with different functional finishings shows a variation of tensile parameters, which indicates that finishing affects the tensile properties and hence the tactile properties of textile fabrics.

Clothing materials usually subjected to a biaxial tensile strain during wearing. This happens because the fabrics are continuously touching the bowed human body and the wearer is always in dynamic motion. Therefore, it is more pertinent to observe the effect of biaxial strain on the tactile properties of the functional fabrics. Changes in the tensile properties of the functional fabrics under low-load regions are shown in Figure 3a. Some of the tensile characteristics are drawn with their logarithmic values for the sake of the scale. Investigations of the tensile behaviors of the fabrics, counting tensile energy (WT), extensibility (EMT), tensile resilience, and linearity (LT) are very vital as textile-based products are indebted to miniature extension and loosening during wear.

As showed in Figure 3a, the changes in the tensile resilience (RT) properties on account of functional finishing treatments were insignificant which is correspondingly supported by the box plot (Figure 3b). This designates that the energy absorption due to elastic deformation and release of the absorbed energy when get rid of the load was not altered due to the various finishing techniques.

A higher value of LT specifies better tensile strength but stiffer property. The tensile linearity LT of the treated samples influenced owing to functional finishing treatments except Sample 4 where inkjet-printing of PEDOT-PSS was executed on a substrate. All other samples become tougher when compared to the control sample (Sample 7). This suggests a modification of the tactile properties of the samples due to the addition of finishing chemicals.

EMT is the fractional addition to a realistic load of 500 gf/cm and has a correlation with fabric’s handle. WT would be characterized by the amount of energy needed for extending the fabric without detrimental destruction. WT notices the toughness of the fabric which designates the textile product under elongation. As showed in Figure 3a, the EMT and WT values of the control sample (7) were found to be ~2.29% and ~5.12 gf.cm/cm^2^, respectively. These values were dropped to ~1.21% and ~3.32 gf.cm/cm^2^, respectively, when the same fabric was treated with photochromic dyes and varnish using inkjet-printing method (sample 1). This articulates that the handle of the fabric can be altered in an uncomplimentary trend. On the other hand, the values changed to ~2.61% and ~5 gf.cm/cm^2^, respectively, when the fabric treated with combinations of PEDOT-PSS, glycerol, and water using inkjet-printing technology (sample 4). The result showed that the handle of Sample 4 was unchanged even if the EMT enhanced. This is ascribed to the handle of the functional fabric might be affected negatively or positively when treated with different chemical reagents and finishing techniques.

The significant tactile property change occurred when the fabric was treated with photochromic dyes. This could be attributed to the nature of the photochromic dye and varnish; probably owing to use of harsh post-finishing parameters (UV-drying-curing procedure). The finishing chemicals were incorporated between the warp and the weft yarns cause to impede the opening between them occupied by the chemicals. The changes in the handle of the other functional fabrics are possibly due to the chemical components used as well as the finishing method.

Figure 3b shows the box plot distribution of the tensile properties at a glance. The box plot showed that the statistical differences and the deviations of each dataset from the median value. Take, for instance, tensile energy WT, most of the dataset fall within the lower quartile (25%); there are few values within the minimum of the median and the others are within the median values. No values recorded within maximum, minimum, or in an outlier (score outside the range). One way or the other, the box plot distribution affirms that the functional finishing might bring a change in the tactile properties of the fabrics as each sample fabric is treated with under different treatment parameters and conditions.

#### 3.2.2. Change in Shearing and Bending Properties

**Figure 4:**
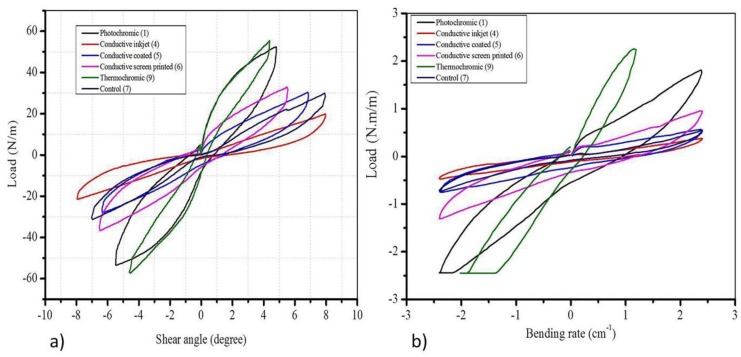
The change in the mechanical characteristics on different functional fabrics under mechanical properties of (**a**) shear and (**b**) bending obtained using KES. The maximum shear angle was 8 degrees. The control sample (7) was used as a substrate and used to produce functional fabrics using different techniques.

**Shearing:** the shearing properties of the functional fabrics were analyzed to observe the effects of the functional finishing on this feature. The measured stress at different shear angle on functional fabrics using similar substrate material but numerous functional treatments is shown in Figure 4a. The obtained result evidently revealed that all types of functional treatment greatly influenced the stress required at each shear angle. The maximum shear angle applied was 8 degrees.

As shown in Figure 4a, for the control sample, the maximum load counts 23.70 N/m at ~7.78 degree shear angles. The conductive sample produced using inkjet-printing showed a distinct trend in load–shear angle relationships. The increase in amount of load for each shear angle occurs very slowly and the maximum load obtained was 19.87 N/m at ~7.95 degree shear angles. The maximum difference was noted between the control and the thermochromic samples. For the thermochromic sample, the maximum load was 55.30 N/m at ~4.38 degree shear angles which is stiffer by 50% than the control sample at the same shear angle. This can be exemplified as the thermochromic sample acquires a higher resistance for deformation due to the addition of stiff thermochromic dyes.

A photochromic sample follows an identical trend to that of the thermochromic sample (52.3 N/m at ~4.8 degrees). Other samples can be tailored and interpreted in a similar approach of interpretation. The greater the resistance for deformation is, the lower the resiliency of the product. This implies that other samples are less resilient than that of the conductive inkjet-printed and control samples. This result assured that the conductive inkjet-printed sample acquires a superior tactile property when compared to other functional fabric samples from the perspective of shearing behavior. This infers that a functional finishing technique, a different application method, and utilizing different chemical reagents possibly influence the tactile properties of fabrics. Therefore, in the course of manufacturing functional and smart textile fabrics the tactile property should not be ignored.

**Bending:**Figure 4b shows the amount of load required to bend the fabrics at different bending rates. The drift with regards to the bending properties of the control, conductive inkjet-printed, conductive-coated, and conductive screen printed samples showed a slight difference exclusively. Therefore, the bending behaviors of these samples follow a similar tendency towards the bending actions. This could be attributed to the demand for similar basic chemical reagents, i.e. PEDOT-PSS. In addition, the aforementioned samples have no larger deviations to the control sample indicating that bending properties were influenced by a smaller amount when fabrics were treated with PEDOT-PSS using various finishing methods. However, thermochromic and photochromic samples show minor fluctuation in bending behaviors than other samples. For example, at a bending rate of 1 cm^−1^, the amount of load inevitable to bend the control, photochromic, and thermochromic samples were ~0.160, ~0.871, and ~2.10 N.m/m, respectively. This demonstrates that the bending resistance increased more than 5-fold and 13-fold compared to the fabric treated with photochromic and thermochromic dyes, respectively. Bending is linked to the stiffness of the fabric and hence the drapability of the cloth produced from the photochromic and thermochromic fabric may be altered. Therefore, tactile properties were slightly influenced when thermochromic and photochromic dyes employed. This could be ascribed to the types of chemical and treatment types during functional fabric finishing.

**Figure 5:**
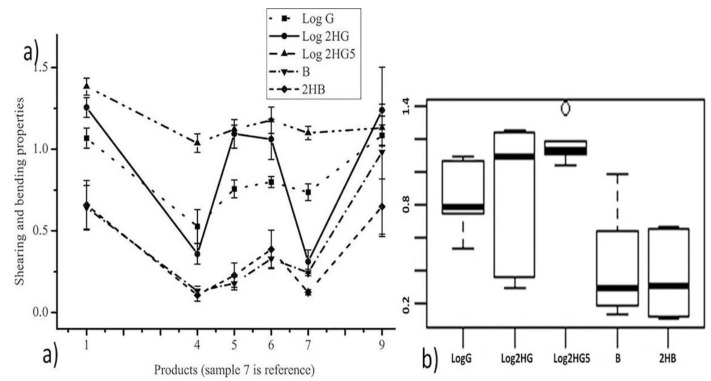
(**a**) The shearing and bending properties results under various finishing methods and (**b**) the box plot showing the variability of the bending and shearing properties within the samples. Some parameters are drawn using logarithmic values for the scaling purpose only. The change in shear hysteresis 2HG for different functional treatment is high when compared to the control sample except in conductive inkjet-printed (4) samples.

The alterations in the shearing and bending properties of the functional fabrics owing to various finishing methods obtained by KES measurement are illustrated in Figure 5a. Measurement of shearing properties for the functional fabrics including shear stiffness (G), shear hysteresis at 0.5°, and shear hysteresis at 5° was performed. Fabric handle and drape depends on shearing properties. The lower the shear parameters are the higher the drapability property of the fabric, and as a result, high tactile properties can be obtained. Shearing rigidity (G) and the shearing hysteresis (2HG & 2HG5) increased quite notably except in the inkjet-printed specimens (sample 4). This could probably be the chemical agglomeration induced when PEDOT-PSS, glycerol, and water introduced into the polyester fabric is fairly low. For instance, Sample 1 appeared to have the lowest resiliency amongst the samples by showing a 2HG value of 18.15 gf/cm more than, ~9 times to that of the control sample and ~8 times to that of Sample 4. Actually, it is the inkjet-printed-conductive sample that contributes to this high resiliency which is nearly equal to the control sample. These changes could perhaps be illustrated as shown in Figure 5b using the box plot distribution chart where most of the values are within the lower quartiles (25%); only a few values are within the upper quartiles in the case of 2HG. This confirmed that there are deviations in the tactile properties of the samples in terms of shearing rigidity due to functional finishing treatments. Furthermore, at an in shear hysteresis value of 5° the box plot showed that there are a few values within in the outlier. The dot in the outlier indicates that there were a few dataset scores which have shear hysteresis outside the normal (median) dataset. Both confirmed each sample recorded different tactile properties in terms of shear rigidity. Other tactile properties can be explained in the same routine.

The increases in the shear hysteresis (2HG) might perhaps be attributed to the uneven surface due to the accumulation of finishing agents. It is obvious that when finishing agents are used to seal the gap between the warp and the weft yarns, rigidity may increase. This is an indication of the influence of finishing methods on the rigidity and resiliency of the fabric. As the resiliency reduced, simultaneously, the tactile properties of the product induced. All the results contributed to draw a conclusion that inkjet-printing produced a preferable product as long as tactile property is concerned.

Bending which is characterized by the resistance when bent is dependent on the friction between yarn and fibers in the fabric structure and the finishing type applied on as claimed in [21]. Therefore, bending is linked to the stiffness of the fabric which is a primary handle property. The bending moment B and bending hysteresis 2HB increased quite noticeably except in the inkjet-printed sample (sample 4). A larger value of B and 2HB indicates a greater fabric inelasticity as a consequence, the stiffness and the resiliency of the product reduced. For example, Sample 9 was found to have the lowest softness value and inflexible product amongst all by recording a B value of ~0.98 gf.cm^2^/cm, more than ~4 times that of the control sample, ~1.5 times that of the sample having the least softness (sample 1), and ~7.5 times that of the inkjet-printed conductive sample (Sample 4). This shows that the tactile properties of the textile product might be influenced by the finishing treatments (probably due to the clusters created when the fabric is treated with photochromic and thermochromic dyes) as well as the auxiliary chemicals during finishing. Therefore, in order to have good clothing comfort, finishing type, and finishing chemicals should be assessed.

#### 3.2.3. Change in the Compressional Properties

**Figure 6:**
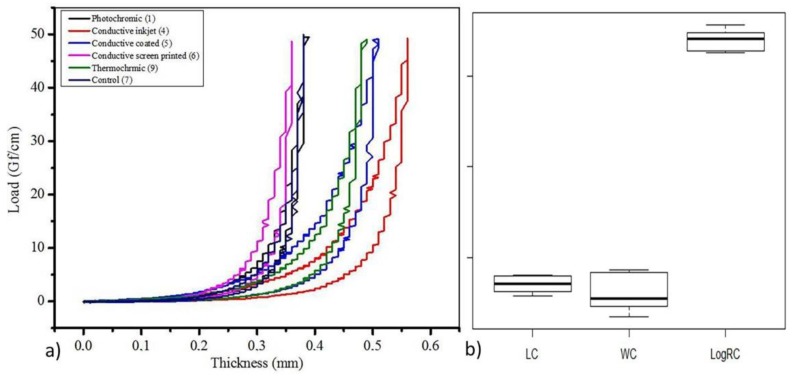
Compressional properties under different finishing conditions; (**a**) the amount of compression load required to compress at a certain fabric thickness and (**b**) the distributions of the compressional datasets in box plot. Fabric thickness is a constructional property measured using KES-3 which is parts that of compressional properties.

Compressional properties of the functional and control fabric such as linearity of compression (LC), compressional energy (WC), and compressional resilience (RC) were measured and analyzed. The compressional changes owing to the different finishing techniques are displayed in Figure 6. Figure 6a shows the amount of load needed to compress the sample at a certain thickness. As showed in the Figure 6a, at ~20 gf/cm the maximum thicknesses for Sample 4, Sample 6, and Sample 7 (control) are ~0.49, ~0.33, and ~0.36 mm, respectively. This conveys that Sample 4 might easily be compressed at higher thickness values with equivalent amount of load when compared to the control sample. On the other hand, Sample 6 requires an equal amount of load to be compressed with lower thickness value when compared to control sample. This shows that Sample 4 is easily compressible compared to the control sample, while Sample 6 is barely compressible than the control sample. The easier the compressibility is, the softer that sample. This could be probably be the lower amount of chemicals present in the inkjet-printed sample so that the gap between the warp and weft yarn is sealed to lesser extent and hence easier to compress.

Figure 6b showed the distribution of the compressional properties measured using KES-3. It is obvious that samples with better compressional properties commonly own maximum LC and WC. As showed in Figure 6b, dataset distribution of the linearity LC of compression for each sample appears to be normal (approximately equal to the median value). This indicates that linearity of the compression has not been much influenced on account of the functional treatment. However, most of the datasets of the work of compression WC are within the upper quartile, even though there is no score outside the outlier. In addition, most of the score of the compression resiliency are within the lower quartiles. This confirmed that compressional energy and resilience were influenced in consequence of the functional treatments. There showed a difference in these two values for each sample. Theoretically, the softer the sample is, the easier it becomes to compress and hence the compressional properties become larger. However, for fabrics processed by various chemicals, this theory seems impractical. This is because the sample becomes easier to compress due to the agglomerated chemical deposited on the surface of the fabrics as the KES machine is quite sensitive to the slight differences happening to the fabric surface. Therefore, the softness reduced as verified by determining the RC values of the samples. RC refers to the recoverability of the fabric after the compressional load detached. The higher the RC value is, the better the recoverability and hence better hand value.

#### 3.2.4. Change in the Surface Frictional Properties

**Figure 7:**
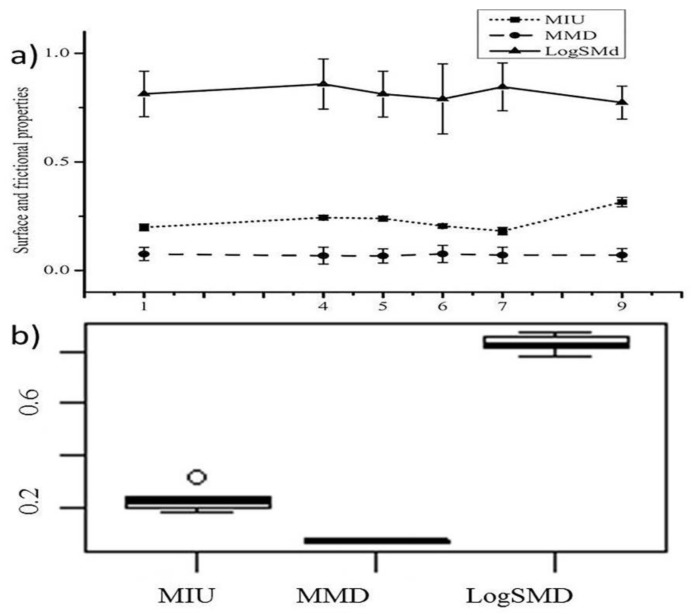
(**a**) The surface frictional properties result under different finishing conditions and (**b**) box plot to indicate the variability within the samples. The surface properties of the functional fabrics were compared with the value of the control fabric.

The changes in the surface and frictional properties of the functional fabrics, which include the coefficient of friction MIU, the geometrical roughness SMD, and the mean deviation of the coefficient of friction MMD, under various finishing methods are shown in Figure 7a.

Take note that the larger the surface frictional values are the rougher the product. As can be seen from Figure 7, an increase in the MIU values of the fabrics occurs after functional treatments. This could be attributed to the increase in surface roughness due to surface or paint imperfections, the adhesion of dirt to the fabric surface, or the presence of contaminated matters. On the other hand, the reduction of the fuzzy fibrils (protruding fibers) when treated owing to the addition of the chemicals and the mechanical actions as mentioned in the work of Kim and Slaten [9] might counteract the increase in surface roughness to have optimum values. Kim and Slaten claimed that the surface irregularities might be masked by the finishing application. All pieces of information guide to appeal a conclusion that MIU can be affected due to functional treatments. This idea is supported by the distribution of the box plot (Figure 7b) where datasets observed on the upper whisker (scores outside the median value) for MIU. This result confirmed that treating the fabric with functional polymers conveyed a change in the surface frictional properties. However, the change in the fluctuation of the frictional coefficient MMD is fairly inconsequential. This means, although the changes are in the smoothness–roughness properties of the samples were significant, the degree of fluctuation in the mean of the friction was negligible.

## 4. Conclusions

In this research, first, the comparative evaluation of low-stress mechanical properties of inkjet-printed, screen-printed, coated, and controlled polyester fabrics from functional finishing point of view were measured and compared. Then, the low-stress mechanical properties of the functional fabrics were interpreted and translated in relation to tactile properties. The result shows that there exists a wide difference in low-stress mechanical properties of different functional fabrics which are related to their tactile properties. Amongst the said samples, inkjet-printing using PEDOT-PSS conductive polymer resulted in worthy tactile properties which on its part might influence the comfort of the cloth produced from it; it acquired superior tactile properties with respect to tensile energy (softness) (WT~5.0 gf.cm/cm^2^), shearing rigidity (G~3.44 gf/cm.deg), compressional softness (WC~0.431 gf.cm/cm^2^), bending rigidity (B~0.131 gf.cm^2^/cm), and surface roughness (SMD~7.43 μm) properties. These results have comparable values to that of the control sample. The result confirmed that it might perhaps be practicable to choose a finishing scheme that could give ideal tactile properties which are the foundations of the quality inspection and evaluation of the functional fabrics. The correlation analysis (up to ~0.99 correlations observed) proved that there existed a virtuous relationship between the quantitative evaluation results obtained by measuring the low-stress mechanical properties of the functional fabrics. The overall findings could be utilized for the selection of suitable finishing parameters and finishing types to attain desirable tactile properties.

## Figures and Tables

**Table 1:** The fabric samples and their production details.

Code	W (gsm); T (mm)	Function	Materials and Chemical Agents	Methods
1	181.8 ± 0.03; 0.62 ± 0.02	Photochromic (K/S; 1.30 ± 0.21)	Reversal Ruby Red dye (Vivimed Labs, Hyderabad, India; 2.5 g L^−1^), dipropylene glycol diacrylate monomer varnish, Ebecryl 81 oligomer (Allnex, Frankfurt am Main, Germany), photo-initiator (Genocure TPO-L; Rahn AG, Zurich, Switzerland)	Inkjet printing 300 dpi
4	186.7 ± 0.15; 0.75 ± 0.02	Conductive (SR; 0.168 ± 0.013 kΩ/square)	PEDOT-PSS (1.3 wt %; Heraeus GmbH, Hanau, Germany), glycerol with water (6:4 *w*/*w*) and Triton surfactant (Sigma Aldrich, St. Louis, MO, USA)	Inkjet printing 300 dpi
5	176 ± 0.2; 0.73 ± 0.01	Conductive (SR; 7.98 ± 0.969 Ω/square)	PEDOT-PSS, DMSO (5%; Sigma Aldrich), U2101 binder (Alberdingk B., Krefeld, Germany), Gel L75N rheology modifier (48 wt %; Borchers, West Lake, OH, USA)	Coating; 200 μm heighst
6	186.8 ± 0.05; 0.56 ± 0.01	Conductive (SR; 4.41 ± 0.396 Ω/square)	PEDOT-PSS, DMSO, U2101, Gel L75N, PET 70 mesh size	Screen printing
9	244.5 ± 0.06; 0.70 ± 0.02	Thermochromic (K/S; 4.63 ± 0.32)	Variotherm AQ ink (5%), ChromaZone extender (95%; Zenit, Stockholm, Sweden), PET 70 mesh size	Screen printing

PEDOT-PSS, poly (3, 4-ethylenedioxythiphene)-poly (styrene sulfonate); SR, surface resistance; DMSO, dimethyl sulfoxide; K/S, color strength; PET, polyester mesh; W and T are weights and thickness of the samples as measured by Kawabata evaluation systems (KES), respectively. Note that all the samples are proposed to produce winter t-shirts for men’s suiting.

**Table 2:** Mechanical and surface properties used by Kawabata [13].

Mechanical Properties	Property	Definitions	Unit
Tensile [KES-1]	EMT	Elongation	%
	LT	Linearity of the curve	-
	WT	Tensile energy	gf.cm/cm^2^
	RT	Tensile resilience	%
Bending [KES-2]	B	Bending rigidity	gf.cm^2^/cm
	2HB	Bending hysteresis	gf.cm^2^/cm
Shear [KES-1]	G	Shear rigidity	gf/cm.degree
	2HG	Shear hysteresis at 0.5°	gf/cm
	2HG5	Shear hysteresis at 5°	gf/cm
Compression [KES-3]	LC	Linearity of Compression	-
	WC	Compressional energy	gf.cm/cm^2^
	RC	Compressional resilience	%
Surface friction [KES-4]	MIU	Coefficient of friction	-
	MMD	Mean deviation of MIU	-
	SMD	Geometrical roughness	μm

**Table 3:** The computed Pearson correlation coefficients between the mechanical properties.

MP	LT	WT	RT	EMT	G	2HG	2HG5	B	2HB	LC	WC	RC	MIU	MMD
WT	−0.87													
RT	0.88	−0.98												
EMT	−0.87	1.00	−0.97											
G	0.86	−0.58	0.70	−0.58										
2HG	0.90	−0.58	0.62	−0.58	0.93									
2HG5	0.83	−0.70	0.72	−0.70	0.77	0.79								
B	0.03	0.37	−0.19	0.37	0.50	0.37	0.00							
2HB	0.59	−0.18	0.32	−0.18	0.89	0.83	0.61	0.77						
LC	0.61	−0.89	0.81	−0.89	0.19	0.23	0.35	−0.67	−0.26					
WC	−0.74	0.53	−0.53	0.53	−0.71	−0.74	−0.81	−0.19	−0.71	−0.17				
RC	−0.47	0.81	−0.72	0.81	−0.04	−0.07	−0.25	0.76	0.41	−0.98	0.01			
MIU	0.12	−0.08	0.20	−0.08	0.29	0.13	−0.33	0.45	0.17	0.10	0.29	−0.07		
MMD	0.64	−0.49	0.48	−0.49	0.60	0.62	0.79	0.11	0.62	0.15	−0.98	−0.01	−0.41	
SMD	−0.91	0.99	−0.97	0.99	−0.63	−0.64	−0.69	0.30	−0.25	−0.87	0.56	0.77	−0.14	−0.50

MP, mechanical properties.
